# Induction of Heme Oxygenase-1 Can Halt and Even Reverse Renal Tubule-Interstitial Fibrosis

**DOI:** 10.1371/journal.pone.0014298

**Published:** 2010-12-13

**Authors:** Matheus Correa-Costa, Patricia Semedo, Ana Paula F. S. Monteiro, Reinaldo C. Silva, Rafael L. Pereira, Giselle M. Gonçalves, Georgia Daniela Marcusso Marques, Marcos A. Cenedeze, Ana C. G. Faleiros, Alexandre C. Keller, Maria H. M. Shimizu, Antônio C. Seguro, Marlene A. Reis, Alvaro Pacheco-Silva, Niels O. S. Câmara

**Affiliations:** 1 Laboratory of Clinical and Experimental Immunology, Nephrology Division, Federal University of São Paulo (UNIFESP), São Paulo, Brazil; 2 Laboratory of Transplantation Immunobiology, Department of Immunology, Institute of Biomedical Sciences IV, University of São Paulo (USP), São Paulo, Brazil; 3 Pathology Division, Federal University of Triângulo Mineiro (UFTM), Uberaba, Brazil; 4 Nephrology Department, School of Medicine, University of São Paulo, São Paulo, Brazil; Inserm, France

## Abstract

**Background:**

The tubule-interstitial fibrosis is the hallmark of progressive renal disease and is strongly associated with inflammation of this compartment. Heme-oxygenase-1 (HO-1) is a cytoprotective molecule that has been shown to be beneficial in various models of renal injury. However, the role of HO-1 in reversing an established renal scar has not yet been addressed.

**Aim:**

We explored the ability of HO-1 to halt and reverse the establishment of fibrosis in an experimental model of chronic renal disease.

**Methods:**

Sprague-Dawley male rats were subjected to unilateral ureteral obstruction (UUO) and divided into two groups: non-treated and Hemin-treated. To study the prevention of fibrosis, animals were pre-treated with Hemin at days -2 and -1 prior to UUO. To investigate whether HO-1 could reverse established fibrosis, Hemin therapy was given at days 6 and 7 post-surgery. After 7 and/or 14 days, animals were sacrificed and blood, urine and kidney tissue samples were collected for analyses. Renal function was determined by assessing the serum creatinine, inulin clearance, proteinuria/creatininuria ratio and extent of albuminuria. Arterial blood pressure was measured and fibrosis was quantified by Picrosirius staining. Gene and protein expression of pro-inflammatory and pro-fibrotic molecules, as well as HO-1 were performed.

**Results:**

Pre-treatment with Hemin upregulated HO-1 expression and significantly reduced proteinuria, albuminuria, inflammation and pro-fibrotic protein and gene expressions in animals subjected to UUO. Interestingly, the delayed treatment with Hemin was also able to reduce renal dysfunction and to decrease the expression of pro-inflammatory molecules, all in association with significantly reduced levels of fibrosis-related molecules and collagen deposition. Finally, TGF-β protein production was significantly lower in Hemin-treated animals.

**Conclusion:**

Treatment with Hemin was able both to prevent the progression of fibrosis and to reverse an established renal scar. Modulation of inflammation appears to be the major mechanism behind HO-1 cytoprotection.

## Introduction

The tubule-interstitial space plays an important role in the progression of chronic renal diseases [1]. Tubule-interstitial changes, including tubular degeneration and interstitial cell infiltration, are a hallmark of common progressive chronic diseases that lead to renal failure [2].

A well-characterized experimental model of renal injury that leads to tubule-interstitial fibrosis is the unilateral ureteral obstruction (UUO). The obstruction of urinary flow leads to release of chemoattractant molecules and growth factors, upregulation of adhesion proteins and degradation of basement membrane matrix proteins [3]. Moreover, a few hours after the obstruction of urine flow, blood-derived macrophages accumulate in the tubule-interstitial space (in healthy renal cortex, the presence of these cells is rare). This cellular infiltration is driven by local expression of molecules such as monocyte chemoattractant protein-1 (MCP-1) and osteopontin [4]. Infiltrating cells release pro-inflammatory cytokines such as TNF-α, IL-6 and IL-1β that activate resident fibroblasts and epithelial tubular cells to initiate the process of epithelial-to-mesenchymal transition (EMT), a process in which polarized epithelial cells undergo biochemical changes and assume a mesenchymal cell phenotype. EMT is associated with release of fibroproliferative molecules, such as TGF-β and PAI-1, and production of extracellular matrix proteins, such as fibronectin and types I and III collagens [5], [6]. The actual challenge is to halt this process in order to avoid end-stage renal failure.

Heme oxygenase-1 (HO-1) is the inducible isoform of the rate-limiting enzyme involved in the degradation of heme. It is responsible for converting heme to carbon monoxide, billiverdin (which is rapidly converted to bilirubin), and free iron (which leads to the induction of ferritin) [7]. HO-1 is strongly induced by oxidative stress and shows cytoprotective effects by the anti-inflammatory, anti-apoptotic, and anti-proliferative actions of its end by-products [8]. Recent studies showed that HO-1 induction is protective in many acute and chronic renal insults [9], [10], [11], [12], [13], [14]. To clarify the role of HO-1 in fibrotic process, Kie *et al* showed that HO-1 knockout mice had significantly greater fibrosis 7 days after UUO compared with wild-type mice, with more extensive EMT [15]. This cytoprotective role of HO-1 was not totally unveiled and may be linked to a modulation of the inflammatory process present in fibrosis.

Recently, some studies have suggested that renal fibrosis could be reversible [16], [17]. Until now, it has been proposed that blockade of the renin-angiotensin-aldosterone system (RAS) is the most effective way to reduce fibrosis [18]. Here, we hypothesize that induction of HO-1, in addition to protecting the kidney from developing fibrosis, can also reverse an established renal scar with improvement in function. Because inflammation is a major component of progression of renal function in chronic renal failure, and HO-1 has evident anti-inflammatory activity, we postulated that this cytoprotection is mainly due to the ability of HO-1 to modulate the immune response.

## Materials and Methods

### Ethics Statement

The animals used in this work were housed in individual standard cages and were kept on a 12 h light/dark cycle in a temperature-controlled room at 21–23°C, with free access to water and food. All procedures were approved by the internal ethical committee of the Federal University of São Paulo (Document n° 0029/2009).

### Animals

Male Sprague-Dawley rats, aged 8–12 weeks (300–350 g), were purchased from Federal University of São Paulo and State University of Campinas Animal Facility Center.

### Drug

Hemin was purchased from Frontier Scientific (Logan, USA), and was dissolved in 0.5 mol/L NaOH, titrated to pH 7.4 with HCl, and made isotonic with distilled water and NaCl. The solution was prepared in darkness just before use and protected from light. Animals were treated with 10 mg/Kg by intraperitoneal (i.p.) injection, according to the protocol described below.

### Experimental model of unilateral ureteral obstruction (UUO)

Rats were briefly anesthetized with Ketamine–Xylazine (Agribrands do Brazil, São Paulo, Brazil) unless otherwise stated. On day 0, UUO was performed by complete ligation of the left ureter with 4-0 silk at 2 points, and an incision made between the points of ligation. Animals were placed in single cages and warmed by indirect light until completely recovered from anesthesia. After recovery, animals were kept under normal housing condition until sacrifice.

### Study design

To check the protective effect of HO-1 upregulation, animals subjected to UUO were either treated or not treated with Hemin. The Hemin was administered at 10 mg/Kg 2 days before surgery. In these studies, animals were sacrificed 7 and 14 days after surgery.

To investigate the capacity of HO-1 in reversing the renal fibrosis, animals were again subjected to UUO and treated with 10 mg/Kg of Hemin i.p. on days 6 and 7 after the surgery. Animals were sacrificed 14 days after the obstruction.

Control animals were subjected to the surgical procedure without ureteral ligation (sham-operated animals). In each study, 10 animals were used for each timepoint assessed.

### Renal Function Outcomes

Blood was collected for serum creatinine measurements, and kidneys harvested for histological, protein and gene expression analyses. Animals were placed in metabolic cages for 24-hours to collect urine. Urinary protein/creatinine ratios were measured in samples collected from the obstructed pelvis and from the bladder at 7 and 14 days post-surgery. All samples were analyzed by colorimetric assays using commercially-purchased kits (Creatinine and Sensiprot Kits, Labtest, Minas Gerais, Brazil). To estimate the urinary albumin concentration, 10 µl of urine were diluted in sample buffer v/v, run on a 10% SDS-polyacrylamide electrophoresis gel and then stained with commassie blue. The gel was photographed using the software GeneSnap (Syngene, Frederick, USA), and quantification was performed by Gene Tools (Syngene, Frederick, USA).

To performe the clearance studies, each designated animal was anesthetized intraperitoneally with 50 mg/kg body weight (BW) of sodium thiopental. The trachea was cannulated with a polyethylene (PE)-240 catheter, and spontaneous breathing was maintained. To control mean arterial pressure and allow blood sampling, a PE-60 catheter was inserted into the right carotid artery. To collect urine samples, a suprapubic incision was made, and the urinary bladder was cannulated with a PE-240 catheter. After the surgical procedure, a loading dose of inulin (100 mg/kg BW diluted in 0.9% saline) was administered through the jugular vein. Constant infusion of inulin (10 mg/kg BW) was then started and continued at 0.04 ml/min throughout the experiment (infusion bomb Harvard Apparatus (Holiston, MA, EUA). A total of three urine samples were collected at 30-minute intervals. Blood samples were obtained at the beginning and end of the experiment. Blood and urine inulin were determined using the anthrone method. Also, blood pressure were measured during clearances experiments through an invasive pressure monitor (Biopac Systems Inc, Aero Camino Goleta, CA, USA), within the carotid artery and kidneys weight were determined after the animals sacrifice. Blood samples were collected during inulin clearance experiments and analyzed with ABL800 FLEX Blood Gas Analyzer (Radiometer America Inc, Westlake, OH, USA).

### Tubule-interstitial fibrosis quantification

Formaldehyde-fixed paraffin sections of the kidneys were stained with Picrosirius. Renal histomorphometric analyses were made by two “blinded” renal histologists. The extent of renal interstitial expansion was quantitatively evaluated in Picrosirius stained sections by a point-counting technique in consecutive microscopic fields at a final magnification of 100X under a 176-point grid with the help of the KS300 program (Zeiss). For Picrosirius staining, 0.1 g of Sirius red and 100 ml of saturated picric acid solution were used to stain 3 µm- thick paraffin sections. Histological sections were deparaffinized with xylol and subsequently hydrated with absolute alcohol (4X) and running water. The sections were then immersed in saturated picric acid solution for 15 minutes and then in Picrosirius for 20 more minutes. Counter-staining was carried out with Harris hematoxylin. Picrosirius stained sections were analyzed by an Olympus BX50 microscope with an Olympus camera attached. Manual shots were taken of the cortex, magnified 40X, and observed under polarized light. Photos of at least 5 different fields in each slide were taken, and structures such as the glomeruli, subcapsular cortex, large vessels and medulla were excluded. The pictures were digitalized (HP Scanjet 2400) and then the interstitial volume of collagen in the cortex compared to the overall cortex area was quantified by morphometry.

For the morphometric analysis, the Image Processing and Analysis in Java, Image J software was used. The result of the analysis is represented by percentage, and refers to the proportion of the interstitial volume of collagen in the cortex to the total cortical interstitial volume, and then the arithmetic mean of the analyzed fields was calculated for each slide. Assessment of the interstitial fibrosis volume obtained by morphometric analysis of the digital image stained with Picrosirius was based on previous studies [19].

### Elisa Assay for TGF-β

Total TGF-β1 protein was measured by ELISA (TGFβ1 Emax®, Promega, Madison, USA). Kidney cells were lysed in RIPA buffer and protein levels quantified by DC Protein Assay (Bio-Rad, Hercules, USA). After overnight coating of a 96-well plate with a primary antibody, TGF-β1 was detected in cell lysates using a secondary antibody. The system uses horseradish peroxidase-conjugated secondary antibody and a single-component TMB substrate for the final chromogenic detection of bound TGF-β1. Using this assay, biologically active TGFβ-1 in tissue culture supernatants, plasma, serum or urine can be detected in the range of 15.6–1,000 pg/ml. Results are expressed as ng/mg of TGF-β protein.

### Gene Profiles

Kidney samples were snap-frozen in liquid nitrogen. Total RNA was isolated from kidney tissue using the TRIzol Reagent (Invitrogen, Carlsbad, Calif) and protocol according to Invitrogen. RNA concentrations were determined by spectrophotometer readings at absorbance 260 nm. First-strand cDNAs were synthesized using the MML-V reverse transcriptase (Promega, Madison, Wisc). RT-PCR was performed using the SYBR Green real-time PCR assay (Applied Biosystem, USA) for the following molecules: Hypoxanthine Guanine Phosphoribosyl Transferase (HPRT) (sense) 5′-CTC ATG GAC TGA TTA TGG ACA GGA C-3′, (antisense) 5′-GCA GGT CAG CAA AGA ACT TAT AGC C-3′; MCP-1 (sense) 5′-AAG AGA ATC ACC AGC AGG T-3′, (antisense) 5′-TTC TGG ACC CAT TCC TTA TTG G-3′; Collagen type I (sense) 5′-TGG CCA AGA AGA CAT CCC TGA AGT-3′, (antisense) 5′-AGA TCA GGT TTC CAC GTC TCA CCA-3′; Collagen type III (sense) 5′-ATG AGC TTT GTG CAA TGT GGG ACC-3′, (antisense) 5′-ACT GAC CAA GGT AGT TGC ATC CCA-3′; PAI-1 (sense) 5′-GAC TGA CAT CTT CAG GTC AAC CC-3′, (antisense) 5′-TCA CCT CGA TCT TGA CCT TTT GT-3′, Catalase (sense) 5′ CAC CAT AGC CAG TGC TCT GC 3′, (antisense) 5′ TGG CAA TGT TCT CAC ACA GG 3′ and TGF-β (sense) 5′-TCA GTC CCA AAC GTC GAG GT-3′, (antisense) 5′-GCT GTG CAG GTG TTG AGC C-3′. RT-PCR was performed using the Taqman real-time PCR assay (Applied Biosystem, USA) for the following molecules: HPRT (Rn01527838_g1), TNF-α (Rn99999017_m1), IL-1β (Rn00580432_m1), IL-6 (Rn00561420_m1), HO-1 (Rn00561387_m1 and Hs01110250_m1), HIF-1α (Rn00577560_m1), Fibronectin (Rn00569575_m1), α-SMA (Hs00426835_g1), E-cadherin (Hs01023894_m1) and GAPDH (Hs03929097_g1). Cycling conditions were as follows: 10 minutes at 95°C followed by 45 cycles at 20 seconds each at 95°C, 20 seconds at 58°C, and 20 seconds at 72°C. Analysis used Sequence Detection Software 1.9 (SDS). mRNA expression was normalized to HPRT or GAPDH expression.

### Western blot Analysis

Kidney cells were lysed in RIPA buffer, run on a 10% SDS-polyacrylamide electrophoresis gel and transferred onto a nitrocellulose membrane (Hybond C Extra, Amershan Biosciences, Little Chalfon, USA). Membranes were incubated with primary goat anti–rat HO-1 (Sigma) antibody, using manufacturer-recommended dilutions, followed by a peroxidase-conjugated mouse anti-goat IgG antibody (Jackson ImmunoResearch Laboratories, WestGrove, USA). HRP activity was detected using enhanced chemiluminescence. The membrane was stripped and probed with mouse primary anti–β-tubulin or anti-β-actin antibody (Sigma, St. Louis, USA) to confirm and estimate the loading and the transfer. We used the software, GeneSnap (Syngene, USA) and Gene Tools (Syngene, USA), to analyze the bands.

### Immunohistochemistry (IHC)

Localization of type I collagen (diluted 1∶300; Abcam, USA) and FSP-1 (diluted 1∶600, A5114, DAKO, Ely, UK) were assessed in paraffin-embedded tissue sections. As described previously, the slides were deparaffinized, rehydrated and subjected to a Tris-EDTA (pH 9) antigen retrieval solution at 95°C. The endogenous peroxidase activity was blocked with 3% hydrogen peroxide, and sections were additionally blocked with Protein Block Solution (DAKO). Slides were incubated with a primary antibody or isotype non-specific IgG as negative control reagent, followed by incubation with the labeled polymer Envision (DAKO), using two sequential 30-minute incubations at room temperature. Staining was completed by a 1–3 minute incubation with 3,3′-diaminobenzidine (DAB)+ substrate-chromogen, which stains the specific antigen brown. Hematoxylin counterstaining was also done.

### Bioplex

Kidney cells were lysed in RIPA buffer with protease inhibitor. A Bio-Plex rat Plex cytokine assay kit (Bio-Rad Laboratories, Inc., Hercules, CA, USA) was used to test samples for the presence of 6 cytokines. The assay was read on the Bio-Plex suspension array system, and the data were analyzed using Bio-Plex Manager software version 4.0. Standard curves ranged from 32,000 to 1.95 pg/mL.

### Cell Culture

HK-2 cells were purchased from American Type Culture Collection (ATCC No. CRL-2190, Manassas, VA, USA). Cells were routinely cultured on 10-cm culture dishes from Sarstedt (Manassas,VA, USA). In the culture medium DMEM-F12 (GIBCO, Invitrogen, Lofer, Austria) was added 100 U/ml penicillin, and 100 µg/ml streptomycin (GIBCO). Cells were fed three times weekly and subcultivated by trypsinization when near confluence. Cells were trypsinized and seeded onto 6-well plates. Cells were grown to 80% confluence before treatment to best represent the in vivo situation of a differentiated epithelial monolayer.

The drugs stock was diluted immediately before treatment and added to the growth medium. Solvent (NaOH 0,1 M) concentrations were at a maximum of 0.2% in all treatment groups, including controls. TGF-β1 (10 ng/ml) was used. After cultivated for 24 h in culture medium serum-deprived, cells were incubated for 24 hours with Hemin (1, 5, 15 or 50 µM). After that, cells were washed twice with PBS and then submitted to total RNA and protein extraction.

### Statistics

The data were described as mean±S.E.M. Differences among groups were compared using ANOVA (with Tukey post-test) and students t-test. Significant differences were regarded as p<0.05. All statistical analyses were performed with the aid of GraphPad PRISM®.

## Results

### Treatment with Hemin improves renal functional outcome

Initially, we aimed to see whether the treatment with Hemin could induce HO-1 expression. For this purpose, we treated human proximal tubular epithelial cells (HK-2) with different concentrations of Hemin for 24 hours. As observed on Figure S1 (Panels A and B), Hemin markedly enhanced HO-1 protein expression on these cells.

**Figure 1:**
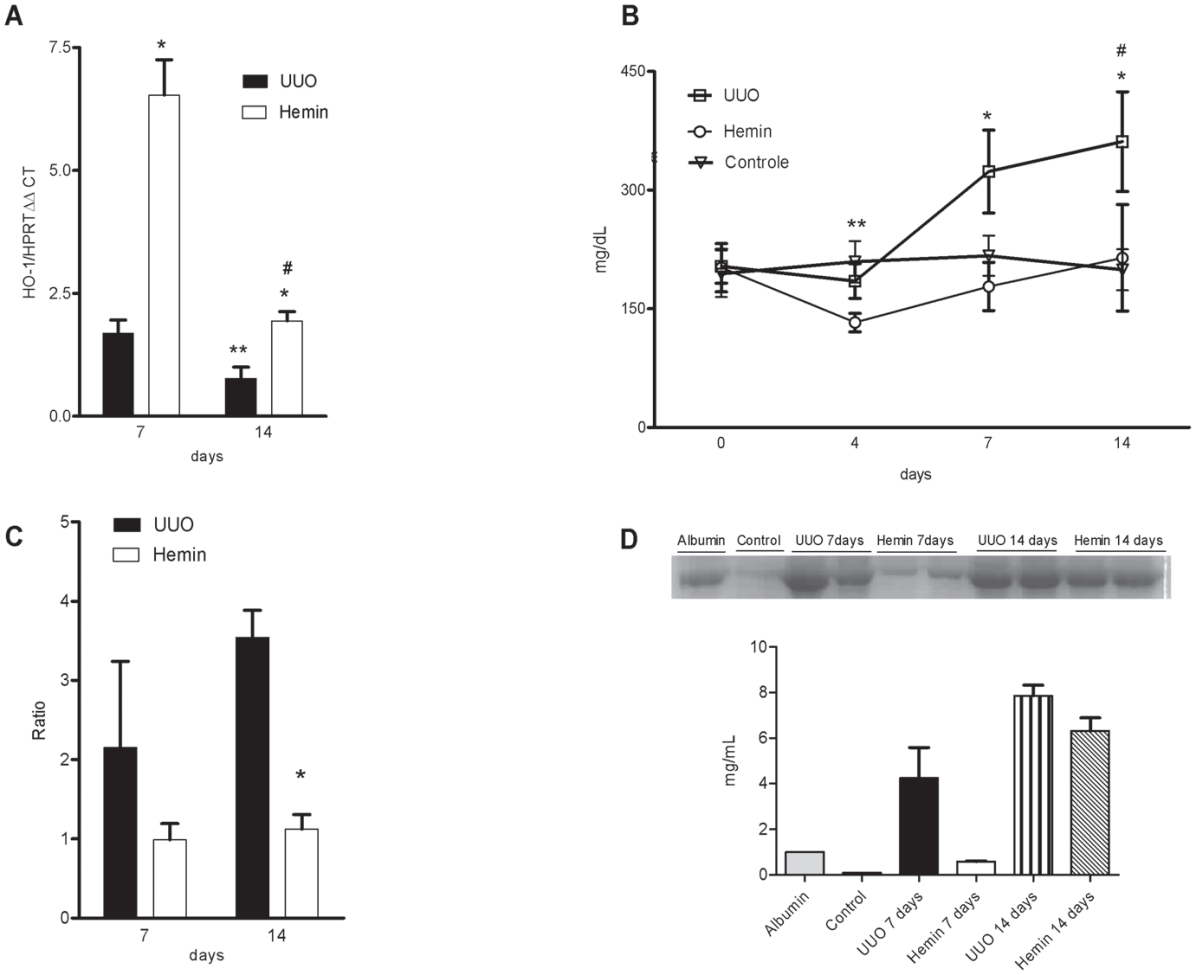
Treatment with Hemin upregulates HO-1 and leads to improved renal functional outcome. (A) Relative mRNA expression of HO-1 in obstructed kidneys from untreated (UUO) and treated (Hemin) animals, 7 and 14 days after surgery. mRNA values from samples are relatively expressed to Control animals, which were assigned a value of 1. * p<0,001 vs. UUO, ** p<0,05 vs. UUO 7 days and #p<0,001 vs. Hemin 7 days. (B) Urine protein values (mg/dL) from bladder of control, UUO and Hemin-treated animals. *p<0,05 vs. Hemin, # p<0,05 vs. Control and **p<0,05 vs. Hemin. (C) Urine protein/creatinine ratio of obstructed pelvis collected by puncture at time of sacrifice from UUO and Hemin-treated animals. *p<0,05 vs. UUO 14 days. (D) Urine albumin excretion analysis. The top panel represents an SDS-PAGE showing the molecular weight of albumin. The graph below expresses the digital optic quantification from the SDS-PAGE. All data are expressed as average ± s.e.m.

Next, we determined whether HO-1 induced by Hemin could indeed protect the kidneys from the development of progressive fibrosis. So, we moved to *in vivo* experiments and the animals were treated with Hemin prior to surgery. UUO induced HO-1 in the kidneys of animals treated with and without Hemin. However, in the Hemin-treated group, there was a significant increase in HO-1 when compared to the untreated group at 7 days after ureteral ligation. After 14 days of surgery, the expression of HO-1 was lower in both groups, but even at that time, there was still greater expression in the treated group (Figure 1A).

**Figure 2:**
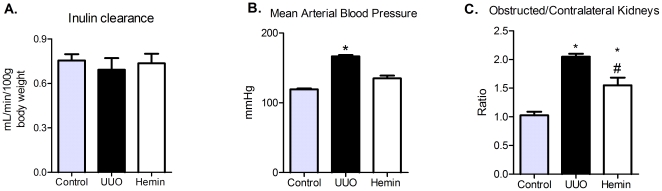
Influence of Hemin treatment on inulin clearance, mean arterial blood pressure and kidneys weight. (A) Inulin clearance was estimated by 100 g of body weight (average ± s.e.m.). No difference between the groups were observed. (B) Mean arterial blood pressure were assessed during inulin clearance experiments (average ± s.e.m.) * p<0,05 vs Control and Hemin. (C) After sacrifice obstructed and contralateral kidneys were removed and weighed. After, a ratio between the kidneys were performed (average ± s.e.m.). * p<0,05 vs Control and # p<0,05 vs UUO.

Once we verified that the treatment promotes upregulation of the HO-1 enzyme, we evaluated the cytoprotective role of HO-1. Serum creatinine levels were not different between the groups (Table 1), also confirmed by inulin clearance measurements (Figure 2A). Moreover, we observed a significant increase in arterial blood pressure in UUO group, compared to control and Hemin-treated animals (Figure 2B).

**Table 1:** UUO surgery does not alter serum creatinine values.

	Control	UUO	Hemin	
*Preview treatment with Hemin*				significance
**Basal**	0,68±0,01	0,66±0,02	0,66±0,04	NS
**14 days**	0,68±0,01	0,68±0,04	0,68±0,02	NS
*Late treatment with Hemin*				
**Basal**	0,60±0,03	0,61±0,05	0,63±0,04	NS
**14 days**	0,60±0,02	0,59±0,02	0,61±0,03	NS

Serum creatinine values (mg/dL) from control, UUO and Hemin-treated animals. Blood samples were collected before surgery (basal) and after sacrifice (14 days).

Serum creatinine values (mg/dL), expressed as average ± s.e.m., from the following groups: control, animals subjected to UUO, and animals subjected to UUO and treated with Hemin. Data indicate values from the pre-treatment protocol, when Hemin was administered before obstruction surgery, and the late treatment protocol, when the drug was administered after established fibrosis.

The obstructed and contralateral kidneys were also weighed and a ratio was calculated. In UUO group, this ratio was significantly higher than in control animals and in Hemin-treated ones (Figure 2C). The metabolic evaluation showed no difference among the groups (Figure S2, panels A–F). We also noticed a marked decrease in proteinuria in Hemin-treated animals, which was evident from the first timepoint until sacrifice (Figure 1B), possibly correlating with arterial blood pressure measurements. Next, we measured the proteinuria present in the obstructed pelvis. As these samples were collected by puncture, we expressed the results by dividing the urine protein levels by the urinary creatinine levels. We observed a lower urinary protein:creatinine ratio after 7 days in the Hemin-treated group, and it was significantly reduced 14 days after UUO (Figure 1C).

**Figure 3:**
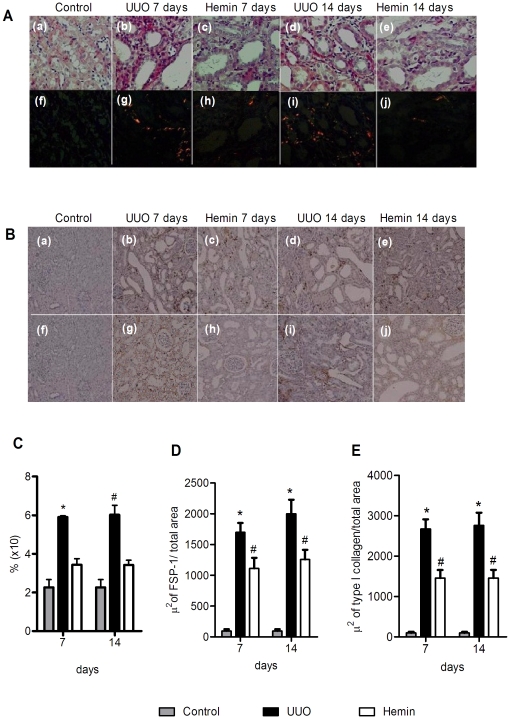
Upregulation of HO-1 promotes less fibrotic tissue deposition in the obstructed kidney. (A) Picrosirius staining from control kidneys and obstructed kidneys of UUO and Hemin-treated animals. Panels a, b, c, d, and e show the tissue when seen using a light microscope and the panels f, g, h, i, and j show the same tissue observed using polarized light. (B) Immunohistochemistry (IHC) for localization of FSP-1 (panels a, b, c, d, and e) and Collagen type I (panels f, g, h, i, and j) from control kidneys and obstructed kidneys of UUO and Hemin-treated animals. (C) Fibrosis area quantification from picrosirius staining (percentage of analyzed field) from groups represented in Figure 2A. The data is expressed as average ± s.e.m. * p<0,05 vs. Control and Hemin and #p<0,001 vs. Control and Hemin. (D) A representative graph showing IHC quantification for FSP-1. *p<0,001 vs Control and Hemin and # p<0,05 vs Control. (E) Representative graph quantification for Collagen type 1 IHC. *p<0,001 vs Control and Hemin and # p<0,05 vs Control.

To check whether the proteinuria observed after surgery was mainly due to the fraction of filtered albumin, we used a 10% SDS-PAGE Coomassie stained gel to indirectly estimate the amount of this protein. The group of animals subjected to UUO that did not receive any treatment had much greater albuminuria than the Hemin-treated animals (Figure 1D). Furthermore, we also examined the extent of interstitial fibrosis by Picrosirius staining, and observed a significantly higher percentage of fibrosis in the untreated group when compared with control and Hemin-treated groups. This difference was more evident on polarized light images, which more clearly show the presence of fibrotic regions on kidney tissue (Figure 3A and 3C). Taken together, these results show that Hemin induced HO-1 upregulation, and the increased expression of this molecule offered renoprotection, as assessed by protein excretion and Picrosirius staining of fibrotic tissue.

### The induction of HO-1 decreases the expression of pro-inflammatory and pro-fibrotic molecules

**Figure 4:**
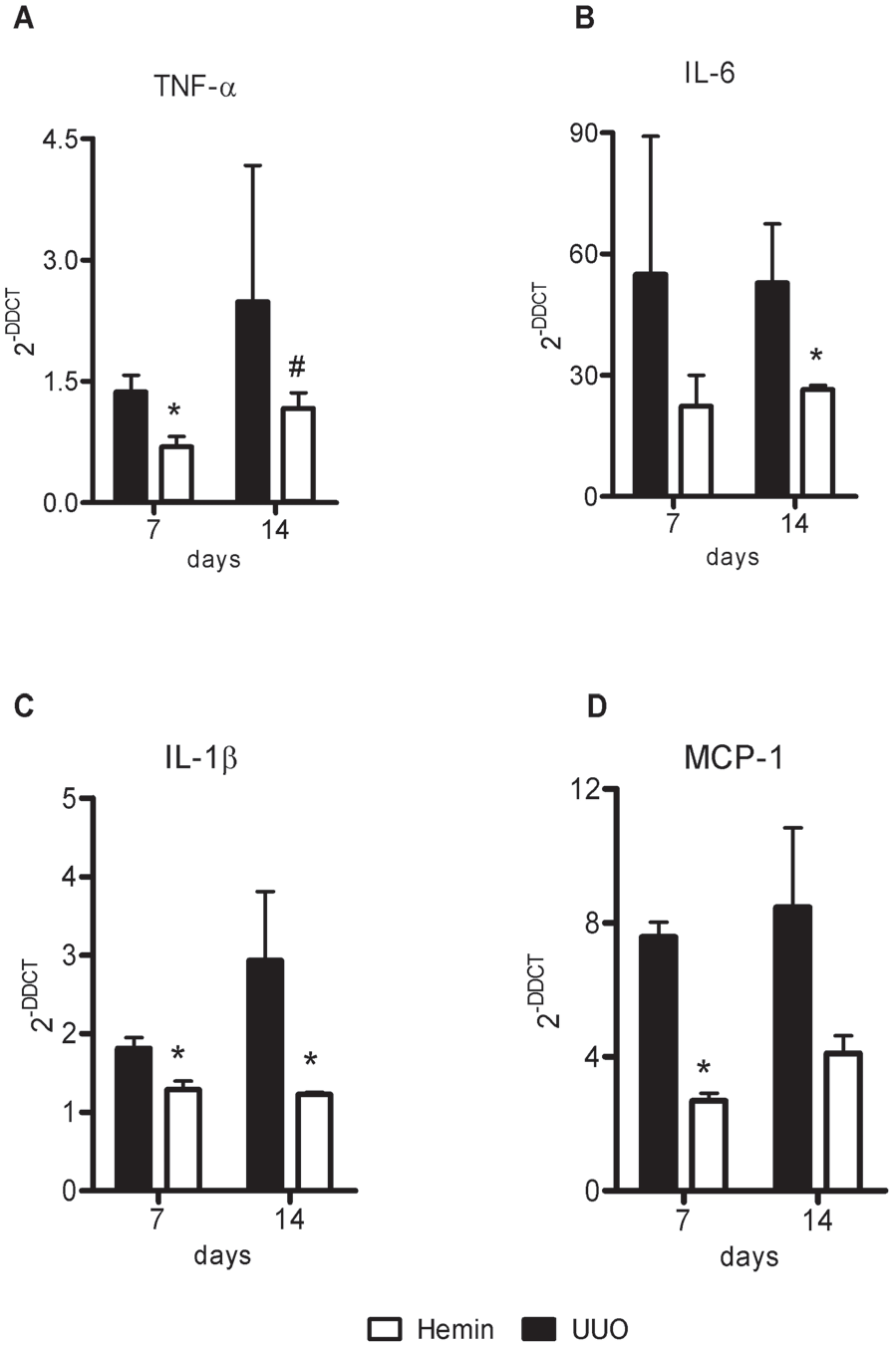
Effects of HO-1 upregulation on inflammatory molecule gene expression after unilateral ureter obstruction. Kidney samples from obstructed kidneys of UUO and Hemin-treated animals were processed and RT-PCR performed for TNF-α (panel A), IL-6 (panel B), IL-1β (panel C), and MCP-1 (panel D). Kidney tissue from normal rats was used as a control, and assigned a value of 1.Data are shown as average ± s.e.m. * p<0,05 vs. UUO and # p<0,05 vs. Hemin 7 days.

**Figure 5:**
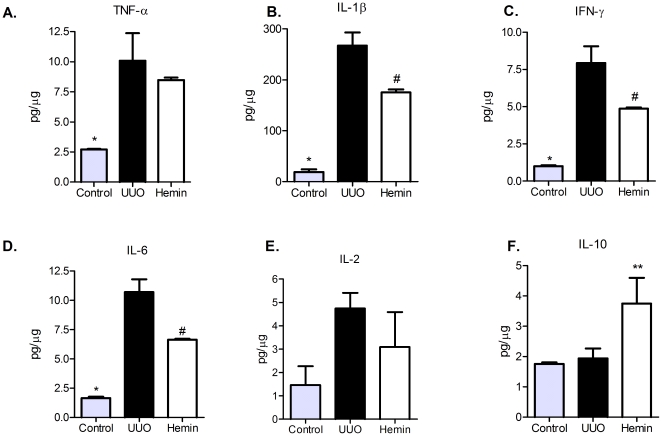
Hemin treatment leads to a decreased kidney protein expression (average ± s.e.m.) of TNF-α (panel A), IL-1β (panel B), IFN-γ (panel C), IL-6 (panel D) and IL-2 (panel E) and enhanced protein expression of IL-10 (panel F). * p<0,05 vs UUO and Hemin, # p<0,05 vs UUO and ** p<0,05 vs control and UUO.

To examine the mechanisms that might be involved in the functional and histological protection observed in Hemin-treated animals, we analyzed the expression of pro-inflammatory and pro-fibrosis-related molecules. In the Hemin-treated animals, there was notably lower expression of TNF-α (Figure 4A), IL-6 (Figure 4B) and IL-1β (Figure 4C). In untreated animals subjected to UUO, there was enhanced expression of MCP-1, but as expected, lower transcription of this chemokine was observed in Hemin-treated animals (Figure 4D). To corroborate these results, we measured the protein expression of some pro- and antiinflammatory molecules. TNF-α, IL-1β, IFN-γ and IL-6 were augmented in UUO group, as well as the lymphoproliferative cytokine IL-2. On the other hand, IL-10 was highly enhanced in Hemin-treated animals (Figure 5, panels A, B, C, D, E and F). We also looked for gene expression of catalase, an oxidative stress marker, and observed that in Hemin-treated group there was a diminished amplification of this molecule, when compared to UUO group (Figure S2, panel G). HIF-1α, a hypoxia marker was also decreased in Hemin-treated group (Figure S2, panel H).

**Figure 6:**
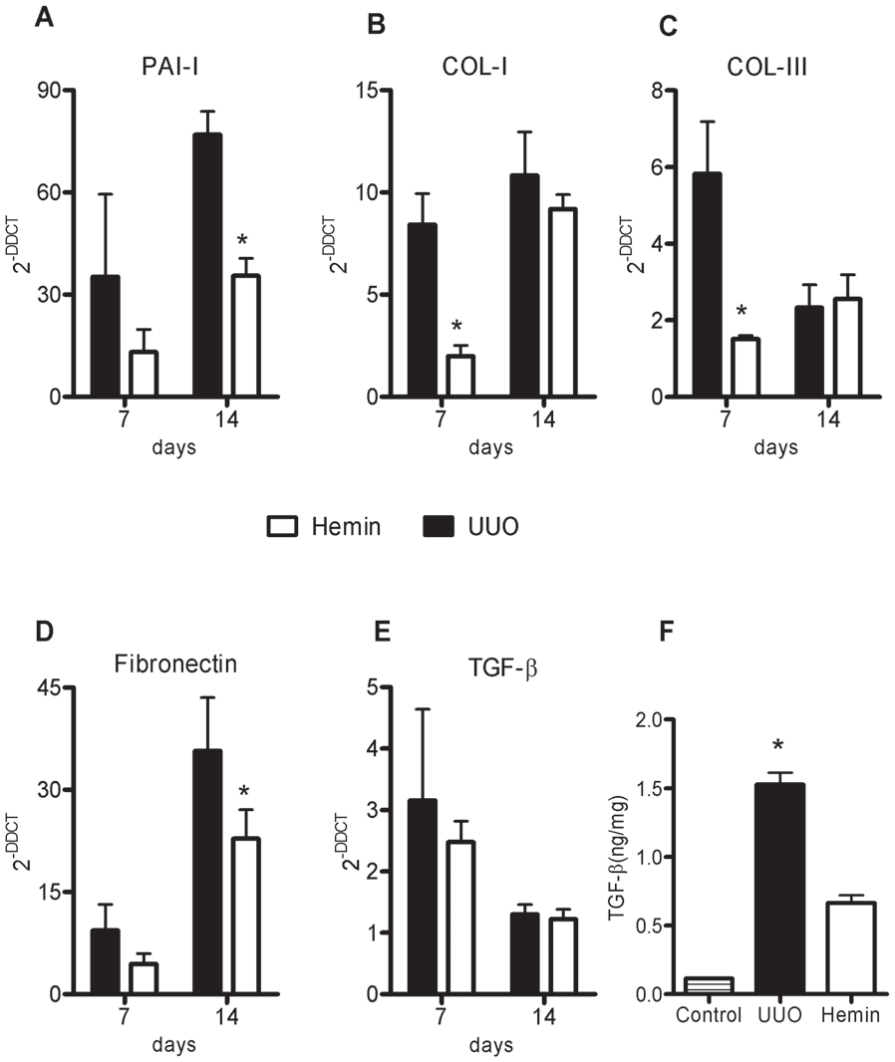
HO-1 upregulation leads to less fibrotic and extracellular matrix gene expression as well as TGF-β protein production. Relative mRNA expression (average ± s.e.m.) of PAI-1 (panel A), collagen types I and III (panels B and C), fibronectin (panel D) and TGF-β (panel E). Samples were obtained from obstructed kidneys of UUO and Hemin-treated groups. Gene expression from control animals was assigned a value of 1. * p<0,05 vs. UUO. (F) TGF-β protein, assessed by ELISA assay, in obstructed kidney samples from UUO and Hemin-treated animals. The amount of TGF-β quantified was divided by total tissue protein. * p<0,05 vs. Control and Hemin.

Next, we examined the gene expression of the pro-fibrotic molecules, PAI-1 and TGF-β, and the extracellular matrix proteins, type I and III collagens and fibronectin. We observed that in Hemin-treated animals, there was lower expression of these molecules, suggesting that HO-1 downregulated pro-fibrotic and extracellular matrix-associated genes (Figure 6, panels A, B, C, and D). Many of these pro-inflammatory and pro-fibrotic molecules were increased after ureteral ligation, but in the Hemin-treated group, this enhancement, when occurred, was more attenuated than in the untreated UUO group. Interestingly, although we noticed a more pronounced expression of TGF-β mRNA in animals subjected only to UUO, when compared to Hemin-treated animals, there was no statistical difference (Figure 6E). However, when we performed the ELISA assay for TGF-β, we observed notably higher protein production of this cytokine in animals subjected only to UUO 14 days after surgery compared to the Hemin-treated group (Figure 6F). Also, type I collagen and FSP-1 (an EMT marker) were enhanced in animals subjected only to UUO, which was not seen in the Hemin-treated group (Figure 3B, 3D, and 3E). To further investigate the mechanisms underlying this process, we performed an *in vitro* assay, where HK-2 cells were treated with Hemin in presence or not of TGF-β. TGF-β is well established inducer of EMT. In fact, the down regulation of α-SMA in TGF-β-stimulated HK-2 cells was prevented in Hemin pretreated cells. Moreover, Hemin did up regulate HO-1 expression while maintained the epithelial phenotype in TGF-β-stimulated HK-2 cells, as seen in E-cadherin expression (Figure S1, panels C, D and E).

These results show that, in the Hemin-treated group, the functional and structural protection observed in conjunction with the upregulation of HO-1 was probably due to modulation of the inflammatory response, which ultimately led to less fibrosis.

### Hemin is able to restore renal function and to reverse fibrosis when renal scars were already established

As we observed the ability of Hemin to prevent the initiation of renal fibrosis, we further asked whether the induction of HO-1 could also reverse established tubule-interstitial disease. To investigate this assertion, animals were treated with Hemin after renal fibrosis was already established.

**Figure 7:**
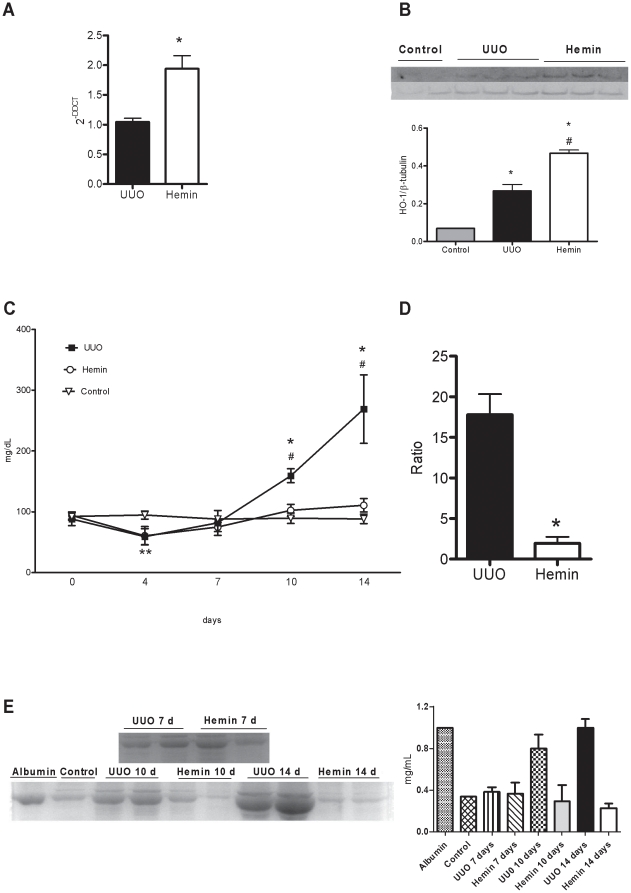
Treatment with Hemin after established fibrosis upregulates HO-1 and improves renal function to that observed in control animals 14 days post-obstruction. (A) HO-1 gene expression, evaluated by RT-PCR, of samples obtained from obstructed kidneys of UUO and Hemin-treated animals. Kidney samples from normal rats were processed and used as control, with mRNA expression assigned a value of 1. * p<0,05 vs UUO. (B) HO-1 protein production as assessed by Western Blotting from control, UUO and Hemin-treated groups. The top panel shows the immunoblot of the HO-1 protein. The samples were also probed with a β-tubulin antibody as a control for the experiment. The graph represents the digital optic quantification of HO-1 protein production divided by β-tubulin protein production. * p<0,05 vs. Control and # p<0,05 vs. UUO. (C) Urine protein kinetics of control, UUO and Hemin-treated groups. UUO and Hemin groups underwent unilateral ureter obstruction and the latter received the HO-1 inducer on days 6 and 7 after surgery. *p<0,05 vs. Hemin, # p<0,05 vs. Control and **p<0,05 vs. Hemin and UUO. (D) Urine protein/creatinine ratio was calculated from the obstructed pelvis of UUO and Hemin-treated animals after 14 days. *p<0,001 vs. UUO. (E) Urine albumin excretion analysis. The left panel represents an SDS-PAGE showing the molecular weight of albumin. The graph expresses the digital optic quantification from the SDS-PAGE. The data are expressed as average ± s.e.m.

**Figure 8:**
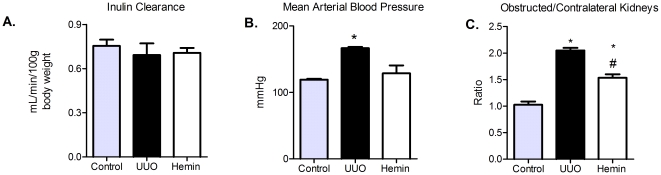
Role of Hemin treatment after 7 days of obstructed nephropathy on inulin clearance, mean arterial blood pressure and kidneys weight. (A) Inulin clearance was estimated by 100 g of body weight (average ± s.e.m.). No difference between the groups were observed. (B) Mean arterial blood pressure were assessed during inulin clearance experiments (average ± s.e.m.) * p<0,05 vs Control and Hemin. (C) After sacrifice obstructed and contralateral kidneys were removed and weighed. After, a ratio between the kidneys were performed (average ± s.e.m.). * p<0,05 vs Control and # p<0,05 vs UUO.

We quantified the mRNA of HO-1 and found that expression of this enzyme was significantly higher in the Hemin-treated group at 14 days after UUO (Figure 7A). HO-1 protein production was also higher in Hemin-treated animals, as shown in Figure 7B. We found no difference in renal function and metabolic evaluation between the groups (Table 1, Figure 8A and Figure S1, panels A–F). However, we found a progressive proteinuria in untreated animals, while the levels of urinary protein were maintained in Hemin-treated animals. When animals were sacrificed, we observed a significant decrease in proteinuria in the bladder and in the obstructed pelvis in Hemin-treated animals compared to animals subjected only to UUO (Figures 7C and 7D). After running the SDS-PAGE for indirect quantification of urinary albumin, we found that, although the initial levels of albuminuria were similar between the groups, Hemin-treated animals showed a significant reduction at 14 days (Figure 7E). Also, untreated animals showed a significantly higher arterial blood pressure and obstructed/contralateral kidneys weight ratio (Figures 8B and 8C).

**Figure 9:**
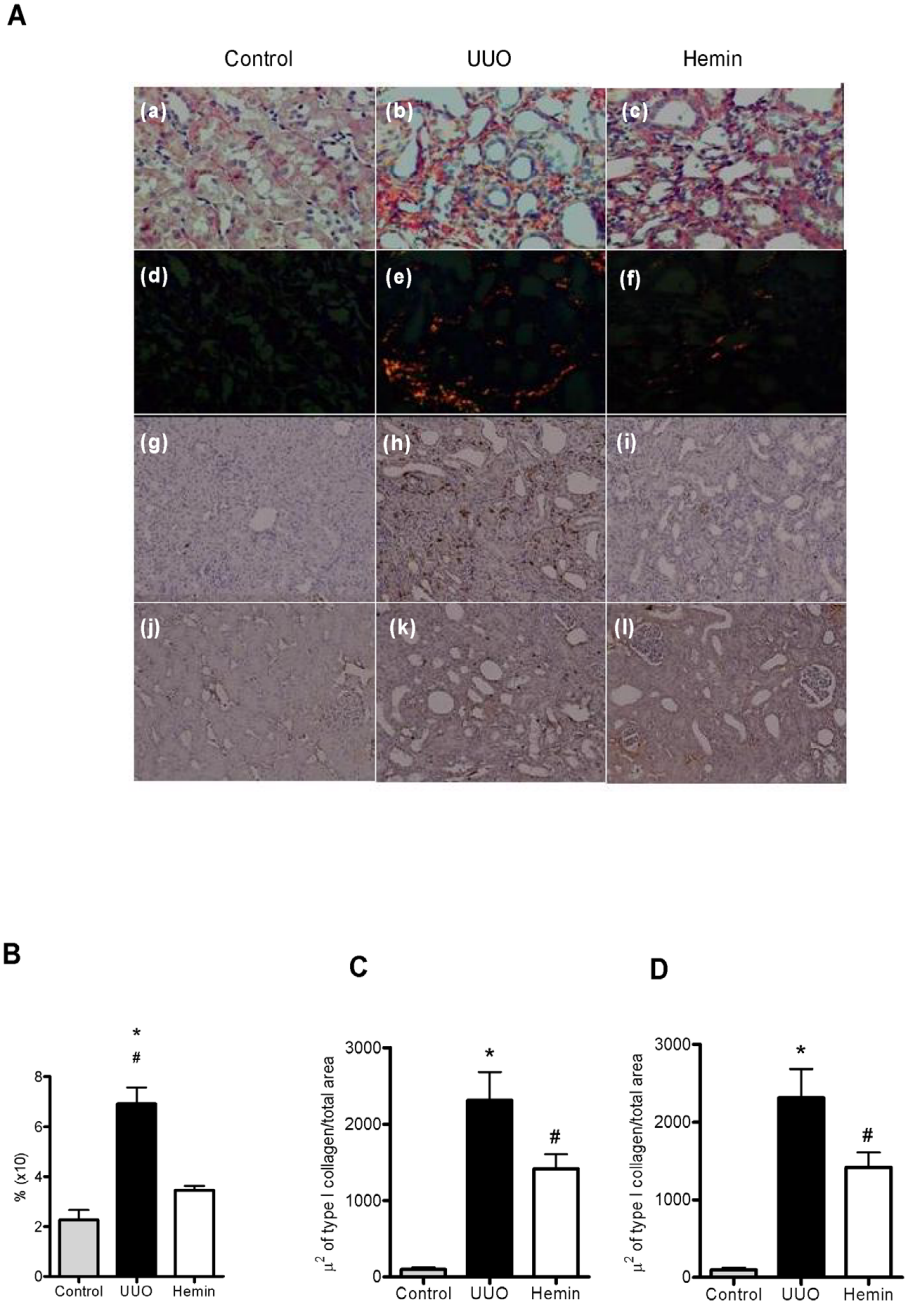
Late treatment with Hemin also diminishes collagen deposition in obstructed kidneys. (A) Picrosirius staining and Immunohistochemistry (IHC) for localization of FSP-1(panels g, h, and i) and Collagen type I (panels j, k, and l) in control, UUO and Hemin-treated groups. Panels a, b, and c represent picrosirius staining, showing strong red staining in the UUO group, which was not seen in control or Hemin-treated groups. The panels d, e, and f show the same image seen using polarized light. (B) Quantification of fibrosis, expressed as average ± s.e.m., from the picrosirius staining shown in Figure 6A. * p<0,05 vs. Control and #p<0,001 vs. Hemin. (C) A representative graph showing IHC quantification of FSP-1. *p<0,001 vs Control and Hemin and #p<0,05 vs Control. (D) IHC representative graph for Collagen type I. *p<0,001 vs Control and Hemin and #p<0,05 vs Control.

Histological analysis also showed a higher percentage of fibrosis in the animals subjected only to UUO when compared to Hemin-treated animals. Indeed, Hemin-treated animals showed an impressive two-fold decrease in the percentage of fibrosis. This striking difference was more evident on Picrosirius staining, especially when viewed as polarized light images (Figures 9A and 9B).

### Reversion of fibrosis by upregulation of HO-1 was associated with a decrease in pro-inflammatory and pro-fibrotic molecule expression

**Figure 10:**
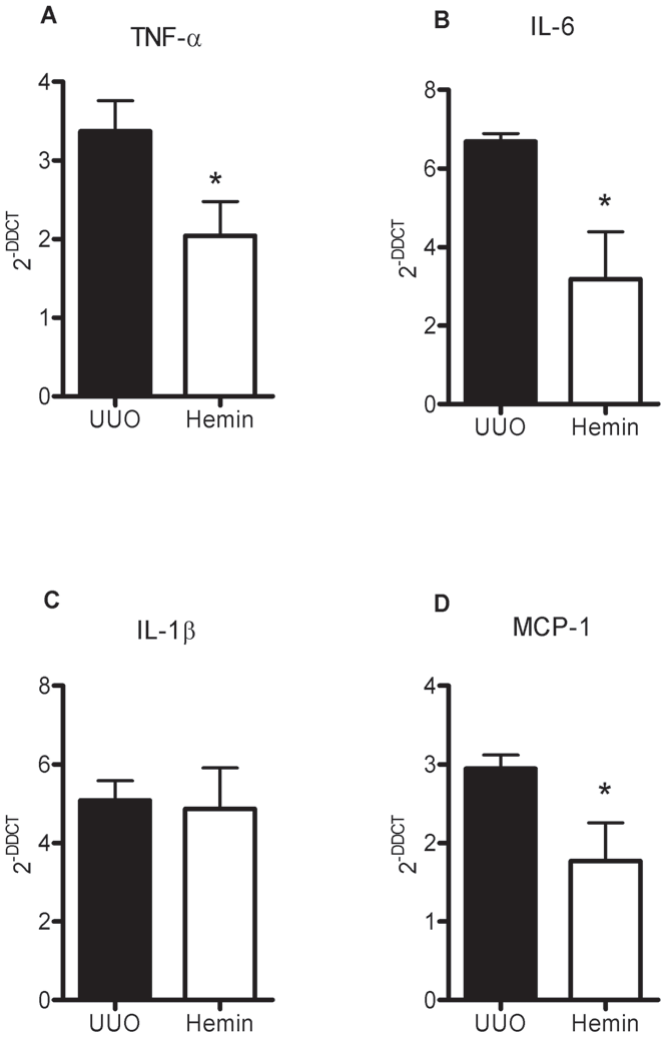
Induction of HO-1 after 7 days of UUO is capable of reducing the signaling molecules involved in the inflammatory process. Gene expression of TNF-α (panel A), IL-6 (panel B), IL-1β (panel C) and MCP-1 (panel D) were assessed by RT-PCR (shown as average ± s.e.m.). Samples were obtained from the obstructed kidneys of UUO and Hemin-treated animals. Renal tissue was collected from animals that did not undergo ureter obstruction surgery and were used as control. Messenger RNA from control animals was assigned a value of 1. * p<0,05 vs. UUO.

**Figure 11:**
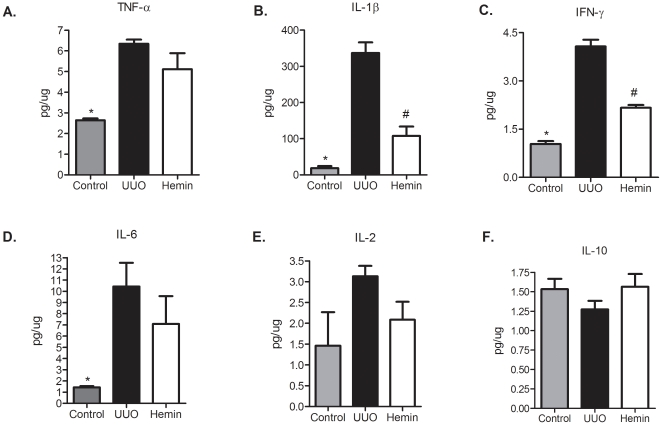
Late treatment with Hemin still maintains a dimished kidney protein expression (average ± s.e.m.) of TNF-α (panel A), IL-1β (panel B), IFN-γ (panel C), IL-6 (panel D) and IL-2 (panel E) and increased protein expression of IL-10 (panel F). * p<0,05 vs UUO and Hemin; # p<0,05 vs UUO.

We next determined whether treatment with Hemin after 7 days of UUO could also reduce the expression of pro-inflammatory molecules. We observed an impressive upregulation of the mRNA of pro-inflammatory cytokines in animals subjected only to UUO. In the treated group, two of the main inflammatory molecules, TNF-α and IL-6, were markedly decreased (Figures 10A and 10B, respectively). The expression of IL-1β was not different between the groups (Figure 10C). As macrophages play a central role in the progression of renal disease, we analyzed MCP-1 expression, which is associated with macrophage chemoattraction to the injured kidney. In the untreated group, we observed a threefold increase in MCP-1 gene expression, and in the Hemin-treated group, we saw a significant reduction in the expression of this molecule (Figure 10D). Catalase and HIF-1α gene expressions were also higher in untreated group (Figure S2, panels I and J). The protein expression of pro- and antiinflammatory cytokines were higher in untreated group (Figure 11, panels A, B, C, D and E). IL-10 was slightly enhanced in Hemin-treated group, when compared to UUO group, but presented similar levels as compared to control group (Figure 11F).

**Figure 12:**
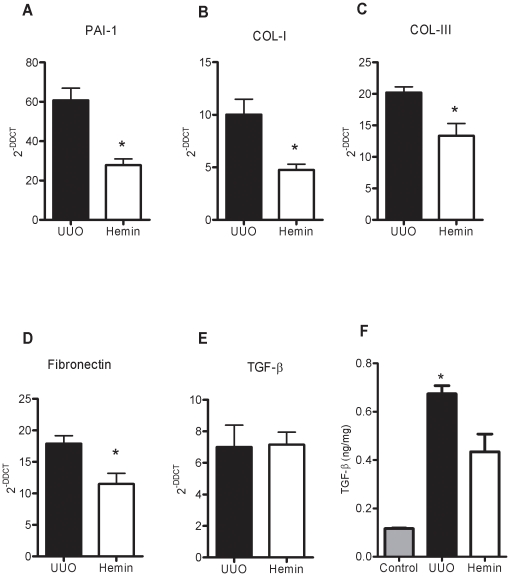
Treatment with Hemin after UUO markedly downregulates gene and protein expression of molecules associated with fibrosis. (A) Samples from obstructed kidneys of UUO and Hemin-treated animals were processed and RT-PCR for PAI-1(panel A), collagen types I and III (panels B and C), fibronectin (panel D) and TGF-β (panel E) was performed (average ± s.e.m.). Gene expression in control animals was assigned a value of 1. * p<0.05 vs. UUO. (F) ELISA assay for total TGF-β protein production in obstructed kidney from UUO and Hemin-treated animals. The amount of TGF-β quantified was divided by total tissue protein. * p<0.05 vs. Control and Hemin.

As an inflammatory state can lead to fibrotic processes, we analyzed the expression of the pro-fibrotic PAI-1 molecule, and observed that treatment with Hemin reduced the expression of PAI-1 when compared to animals subjected only to UUO (Figure 12A). Furthermore, we observed impaired expression of extracellular matrix-related proteins in animals treated with Hemin: expression of fibronectin and types I and III collagen was significantly lower in the Hemin-treated group as compared to those animals subjected only to UUO (Figure 12, panels B, C, and D). Indeed, type I collagen protein deposition, as well as FSP-1, was notably decreased in the Hemin-treated group, as shown in Figure 9.

Although we saw no difference in TGF-β mRNA between the groups (Figure 12E), TGF-β protein production as assessed by ELISA was significantly higher in the untreated group compared to the Hemin-treated group (Figure 12F). These results show that even with late treatment, HO-1 induced a decrease in molecules associated with renal inflammation, as well as molecules promoting fibrosis and extracellular matrix deposition, which ultimately led to renal protection.

## Discussion

In the present study, we tested two hypotheses: first, that upregulation of HO-1, with a non-toxic dose of Hemin, could limit the development of renal fibrosis and second, that HO-1 could also, more importantly, reverse the progression of renal fibrosis, once the scar was already established. Indeed, our data show that upregulation of HO-1 has renoprotective functions in both conditions, and that the mechanisms behind cytoprotection are probably mediated by modulation of the inflammatory response.

The experimental model chosen by us was the UUO, that despite not reproducing all features seen in renal fibrosis in humans, it is fully reproducible and a widely used model [3], [4], [20]. This model allows us to design a strategy where we could treat the animals when scar was already presented and quantified. UUO leads to tubule-interstitial fibrosis in a process involving cellular infiltration, tubular proliferation and apoptosis, (myo)fibroblast accumulation, increased extracellular matrix (ECM) deposition and tubular atrophy. A variety of mechanisms, including immunological ones are involved in these processes. One impressive advantage of this experimental model is that the different features of the pathology appear rapidly, all within around one week after the induction of the pathology, making suitable to perform experimental approaches to halt the scaring process. However, it is noteworthy to emphasize that the UUO model has its known limitations. Some features classically present in CKD in human is less prominent in UUO, which might limit our conclusions. The data provide here should be careful interpreted when considered other models of CKD.

Initially, we observed a significant amplification of HO-1 mRNA in Hemin-treated animals. UUO by itself is capable of inducing HO-1 expression as observed by Moriyama and colleagues [21] due to its ability to respond to cellular stress. Moreover, it can rapidly be induced by exposure to a number of factors, including ultraviolet radiation, hydrogen peroxide, heavy metals, cytokines (IL-6, TNF-α, IL-10), and LPS [22], [23], [24], [25]. Altogether, the inflammatory milieu and cell stress generated by the obstruction might be responsible for the induction of HO-1. Here, in this work, we optioned to induce HO-1 by treating the animals with Hemin. This drug is widely used for this purpose for a long time ago and its cytoprotective roles were recent reviewed [26], [27], [28], [29], [30]. Indeed, Hemin was able to induce HO-1 in our experiment in cells and in animals.

HO-1 has been shown to suppress some cellular processes that participate in fibrosis in different animals models or *in vitro* systems [31]. For this reason, we speculated that HO-1 would be a suitable molecule to investigate in halting the progression of renal disease.

Here, we noticed progressive urinary protein excretion after surgery, as observed in other experimental models of chronic kidney disease [32], [33]. These data demonstrated that the contralateral (unobstructed) kidney also suffers from UUO, possibly due to glomerular hyperfiltration and hypertension. We also observed less urinary albumin excretion in the group treated with Hemin that could be partially associated with the observed reduction in arterial blood pressure.

Our study showed lower expression of pro-inflammatory molecules in the Hemin-treated group. Previous work showed a positive correlation between fibrosis and overexpression of TNF-α, IL-6 and IL-1β in the UUO model [34], [35], [36]. In UUO, there is activation of the renin–angiotensin system with production of reactive oxygen species (ROS) and NFkB, which promote macrophage infiltration [37], renal tubular apoptosis and interstitial fibrosis [38]. It may be due to NFkB that inflammatory cytokines (TNF-α, IL-6 and IL-1β), chemokines (MCP-1, MIP-1 and IL-8) and adhesion molecules (ICAM-1, VCAM-1 and E-selectin) [39] were upregulated, similar to that seen in our studies. Furthermore, activated macrophages can generate TNF-α, which mediates pro-apoptotic signaling that leads to renal tubular cell apoptosis following UUO [20]. HO-1 acts to release antioxidant molecules such as bilirubin and ferritin, which can neutralize the ROS produced mainly by infiltrating inflammatory cells, resulting in less tissue damage.

Renal fibrosis results from an imbalance between synthesis and degradation of extracellular matrix components, with enhanced production of types I and III collagens and fibronectin [40]. In our study HO-1 upregulation decreased collagen deposition and FSP-1 positive cells infiltrate, in accordance to another study which demonstrated that animals subjected to UUO and then exposed to low doses of CO had reduced α-SMA induction and less type I collagen and fibronectin deposition [41]. Here, we cannot rule out the effect of HO-1 on pelvic pressure. Indeed, pelvic pressure could influence the fibrotic state. Previous works stated that on early stages after the onset of UUO, intratubular pressure is threefold elevated, and this reduces to an intermediate level by 24 h. But, sustained increases in tubular pressure are likely in this model, once urine continues to pool as demonstrated by studies in dogs [42], [43].

Many pro-fibrotic molecules were described during renal fibrogenesis. Matsuo *et al* described the role of PAI-1 in interstitial fibrosis [44]. Moreover, Matsumoto *et al* also showed that HO-1-deficient cells expressed higher PAI-1 levels and its significantly reduction after treatment with bilirubin or CO [45]. In our study, PAI-1 was also significantly reduced in Hemin-treated animals, most at later time points. We also investigated the role of TGF-β, since it is the main pro-fibrotic cytokine [46], and previous work showed that inhibition of TGF-β attenuated renal interstitial fibrosis in obstructive nephropathy [47], [48]. TGF-β protein (its active form) production was significantly enhanced in the group subjected to UUO and that received no treatment. Conversely, animals treated with an HO-1 inducer demonstrated significantly less TGF-β expression, as seen by other works [9], [49]. In the *in vitro* assay, the presence of TGF-β enhanced mesenchymal and decreased epithelial markers in proximal renal tubular cells, as observed by others [50], [51]. This situation was abolished when cells were previous exposed to Hemin, suggesting a role for HO-1 in halting EMT process.

However, all the data presented up to now do not address the issue of whether HO-1 could actually reverse established renal fibrosis. In this context, we next evaluated the ability of Hemin therapy administered post-UUO to reduce renal scar formation. Our results clearly show that late treatment with Hemin still led to upregulation of HO-1. The gene and protein expression of this molecule were enhanced even 7 days after the drug were administered, which confirms the effectiveness of the treatment. In fact, the treated group, at sacrifice time, had the same renal functional values as the control group. Furthermore, we observed a striking reduction of inflammatory molecules in the treated group. This is possibly due to the anti-inflammatory properties of HO-1 byproducts. It is important to highlight, however, that late treatment with Hemin was able to reduce the expression and production of pro-fibrotic molecules, as well as type I collagen and FSP-1 deposition on the injured kidney. Together, the results from the delayed treatment with Hemin showed an impressive capacity to reverse progressive renal disease. As patients with chronic kidney disease usually begin treatment after established fibrosis, this study is highly relevant to clinical practice, thus emphasizing its importance.

Studies showing reversion of already established fibrosis have become quite important, as EMT, a pivotal pathway for fibrosis development, has been shown to be reversible. Previous work showed that in a patient with diabetic nephropathy, a pancreas transplant resulted in long-term normoglycemia and led to a marked reduction in mesangial and mesangial matrix fractional volumes [52]. Also, a recent study showed that, in a 5/6 nephrectomy model, delayed treatment with high doses of a type 1 angiotensin II receptor antagonist led to regression of glomerulosclerosis, possibly by inhibiting PAI-1 and modulating extracellular matrix turnover [53].

An *in vitro* study demonstrated that HO-1 upregulation could prevent cyclosporine A-induced EMT via NRF2, a master regulator of genes associated with the cellular antioxidant system [54]. Kie *et al* also showed that HO-1 deficient mice subjected to obstructive nephropathy had increased fibrotic tissue deposition, as well as greater tubular TGF-β1 expression, inflammation, and enhanced EMT [15]. These data corroborate our results and suggest that HO-1 expression is important to attenuate EMT. More importantly, HO-1 overexpression is able to reverse this process.

HO-1 was able not only to protect the kidney from the onset of progressive fibrosis but also, and more importantly, to reverse its progression; HO-1-induced protection was evident in improved renal functional outcome and lower expression of pro-inflammatory and pro-fibrotic molecules. Taken together, we propose that HO-1 induction might represent a promising therapy for limiting chronic kidney disease.

## Supporting Information

Figure S1 HO-1 upregulation is able to modulate fibrosis in vitro. HK-2 cells were incubated with different doses of Hemin for 24 hours and Western Blotting was performed for HO-1 protein expression. Panel A shows the image obtained from the blotting and panel B represents its relative quantification. The same cells were cultivated and treated with Hemin and/or TGF-Î^2^ for 24 hours. After that, mRNA was extracted for later quantification by Real Time PCR for the following molecules: HO-1 (panel C), Î±-SMA (panel D) and E-cadherin (panel E). Gene expression in control cells was assigned a value of 1.
(0.59 MB TIF)

Figure S2 Hemin treated animals present similar levels of serum metabolic parameters than untreated ones, but show decreased oxidative stress and hypoxic environment. The metabolic evaluation is represented by pO2 (panel A), pCO2 (panel B), Hematocrit (panel C), Serum Na+ (panel D), Serum K+ (panel E) and Serum bicarbonate (panel F). mRNA expression of Catalase and HIF-1Î± were performed by real time PCR from animals submitted to UUO and treated or not with Hemin prior to surgery (panels G and H, respectively), or 7 days after the surgical procedure (panels I and J). All animals were sacrificed after 14 days of surgery. Gene expression in control animals was assigned a value of 1.
(0.41 MB TIF)
